# One size fits all? Mixed methods evaluation of the impact of 100% single-room accommodation on staff and patient experience, safety and costs

**DOI:** 10.1136/bmjqs-2015-004265

**Published:** 2015-09-25

**Authors:** Jill Maben, Peter Griffiths, Clarissa Penfold, Michael Simon, Janet E Anderson, Glenn Robert, Elena Pizzo, Jane Hughes, Trevor Murrells, James Barlow

**Affiliations:** 1 Florence Nightingale Faculty of Nursing and Midwifery, King's College London, London, UK; 2 Faculty of Health Sciences, University of Southampton, Southampton, UK; 3 Institute of Nursing Science, University of Basel, Basel, Switzerland; 4 Directorate of Nursing/AHP, Inselspital Bern University Hospital, Bern, Switzerland; 5 Faculty of Population Health Sciences, Institute of Epidemiology & Health, UCL, London, UK; 6 Independent researcher, Winchester, UK; 7 Business School, Imperial College, London, UK

**Keywords:** Nurses, Patient safety, Patient satisfaction, Infection control, Human factors

## Abstract

**Background and objectives:**

There is little strong evidence relating to the impact of single-room accommodation on healthcare quality and safety. We explore the impact of all single rooms on staff and patient experience; safety outcomes; and costs.

**Methods:**

Mixed methods pre/post ‘move’ comparison within four nested case study wards in a single acute hospital with 100% single rooms; quasi-experimental before-and-after study with two control hospitals; analysis of capital and operational costs associated with single rooms.

**Results:**

Two-thirds of patients expressed a preference for single rooms with comfort and control outweighing any disadvantages (sense of isolation) felt by some. Patients appreciated privacy, confidentiality and flexibility for visitors afforded by single rooms. Staff perceived improvements (patient comfort and confidentiality), but single rooms were worse for visibility, surveillance, teamwork, monitoring and keeping patients safe. Staff walking distances increased significantly post move. A temporary increase of falls and medication errors in one ward was likely to be associated with the need to adjust work patterns rather than associated with single rooms per se. We found no evidence that single rooms reduced infection rates. Building an all single-room hospital can cost 5% more with higher housekeeping and cleaning costs but the difference is marginal over time.

**Conclusions:**

Staff needed to adapt their working practices significantly and felt unprepared for new ways of working with potentially significant implications for the nature of teamwork in the longer term. Staff preference remained for a mix of single rooms and bays. Patients preferred single rooms.

## Background

Historically hospital design was based on Florence Nightingale's nineteenth-century observations about the advantages of natural light, ventilation and cleanliness.^1^ Shared patient accommodation of 30 beds became the standard inpatient accommodation in hospitals globally.^2^ The suitability of such wards in modern hospitals is now questioned in terms of quality, safety and experience.^3^ Subdivisions of wards into smaller rooms and bays has become the norm, and internationally, the case is being made for more single-room accommodation in new hospital designs. Arguments for the abolition of all shared accommodation^4^ are based largely on the belief that patients prefer single rooms and benefit from improved patient outcomes compared with open hospital wards.^5^

Most current evidence derives from the USA and Scandinavia,^6^ and while some evidence may be transferable, variation in financial, cultural and organisational systems means that generalisation should not be assumed. The available evidence is both weak and equivocal, suggesting a range of potential benefits for patients and staff but also potential disadvantages.

Potential advantages of single-room accommodation for patients include increased patient privacy, dignity, comfort and less disruption from other patients^7–9^ and also enhanced patient satisfaction with communication with staff.^7–10^ Patients rated privacy and personal space as important but they also said that when ill they wanted nurses to be closer.^11–13^ Potential advantages for staff of working in single rooms include more personalised contact with patients and fewer interruptions for care delivery.^14^ Improved safety outcomes could include fewer medication errors,^15^ faster patient recovery rates^7–9^ and reduced infection rates.^7^ ^16–18^ Perceived economic advantages include the potential for cost savings associated with reduced length of stay and infection rates and fewer medication errors.^19^ ^20^

Potential disadvantages of single-room accommodation for patients include increased rates of slips, trips and falls and other adverse events through less patient surveillance,^7–9^ ^21^ and reduced social interaction^22^ and patient isolation^7–9^ ^23^ with consequent negative impacts on patients’ mental well-being and behaviour.^24^

Suggested disadvantages of single rooms for staff/organisations include an increase in staff walking distances (reducing time for direct care)^25^ and workload^3^; potential need for an increase in staffing levels and/or adjustments to staff skill-mix^8^ ^9^ ^24^; increase in staff stress^26^ ^27^ and potential risks to staff through working in isolation.^26–30^

The international healthcare literature on the advantages and disadvantages of single-room accommodation for patients and staff is of variable quality, and some aspects have been studied more closely than others. Research on the impact of single-room accommodation on staff experiences of work and care provision is limited, the evidence does not clearly point to a preference for single rooms among patients and little is known about patient preferences across different age and cultural groups.^31^ There is very little evidence from well-designed research studies, and most claims are unsupported by evidence of changes in clinical outcomes; research designs are weak, typically relying on staff opinion or simple before-and-after comparisons.

Finally, it is clear that decisions around ward design are complex and trade-offs are likely to be necessary between design considerations, patient and staff preferences, changes in clinical needs/demand and economic considerations.

## Objectives

We aimed to identify the impact of the move to a newly built acute National Health Service (NHS) hospital in England with 100% single rooms on staff and patient experience, patient safety and costs.

## Design and methods

### Study setting

In 2011, Tunbridge Wells Hospital opened, replacing old facilities (some 100 years old) at Pembury and Kent and Sussex hospitals. This was the first NHS hospital in England to have 100% single inpatient rooms in all wards. Staff and patients moved from predominantly large bays and open Nightingale wards in two phases in January and September 2011.

### Design

The study was a mixed methods evaluation of the impact of the move and included a before-and-after study of patient and staff experience, a quasi-experimental before-and-after study of safety outcomes using two control hospitals, and a cost analysis. Data were collected in the new hospital between September 2012 and June 2013. A fuller report of the study and methods can be found elsewhere.^6^

### Patient and staff experience

Trust-wide data comprised 20 pre-interviews and 21 post-interviews with senior managers (eg, director of nursing, of infection control and of therapies), clinicians (doctors, allied health professionals, ward managers), ward clerk and staff managing the move or architects/builders (see online supplementary appendix). Other patient and staff data were collected from four case study adult inpatient wards, purposively selected to encompass a range of different clinical areas and patient groups: medical assessment unit (MAU), surgical, medical (older people) and maternity. These data comprised 119 hours of structured ward observations pre and 250 hours post; 24 pre and post semi-structured ward staff interviews. The observation of practice was to understand how and where staff spent their time and determine whether the proportion of time they spent on each activity changed following the move to single-room accommodation. The researcher shadowed staff and recorded their activities on a personal digital assistant using HanDBase software in a structured time and motion data collection tool developed by the research team. This drew on a similar tool used in healthcare research designed and developed by Westbrook and Ampt^32^ (work observation method by activity timing) (see online supplementary appendix for observation categories). The following data were also collected in each of the four case studies: 32 pre and post semi-structured patient interviews; 55 pre and post staff surveys and pedometer data from 53 staff pre move and 56 post move. Further details can be found in the online supplementary appendix.

The structured observation, pedometer and staff survey data were entered into Excel and analysed in SPSS using descriptive and parametric statistics.^6^ Staff perceptions of the ward environment pre and post move were compared statistically using an unpaired t test. Travel distance data were analysed using a repeated-measures general linear mixed model (SPSS MIXED) with steps per hour as the dependent variable and pre-post new build, ward (maternity, surgical, older people, MAU), observation session (repeated measure), staff group (midwife, registered nurse (RN), healthcare assistants (HCAs)) and day of the week as independent variables. Midwives were only employed on the maternity ward, and therefore, this introduced an element of confounding. For this reason, the maternity ward was excluded from the modelling. Its exclusion resulted in an improved model fit. A sensitivity analysis to assess the effect of repeated measures on the small number of people who contributed more than one data collection session found that there was no effect and so the data were treated as a between-subjects design. The moderating effects of ward and staff group on the pre-post move were also tested by fitting these two interactions to the main effects model. F tests and adjusted means with 95% CIs have been presented.

Interviews were recorded, transcribed verbatim and analysed using a framework approach, a method that involves a systematic analysis of verbatim interview data within a thematic matrix.^33^ Data synthesis occurred as part of the analytical process, and connections were made between qualitative and quantitative data sources in order to identify core themes and connections.^34^ ^35^

### Safety outcomes

We recruited two control hospitals; one in which no move occurred (steady-state control) and one that moved to a new build with an increase in single rooms (but fewer than 100% single rooms) during the study period (new-build control). Patient outcomes (safety events—falls, pressure ulcers, medication errors—and hospital-acquired infections—methicillin resistant *Staphylococcus aureus* (MRSA) and *Clostridium difficile*) were collated for a 3-year period from January 2010 to December 2012 for three of the four wards (MAU, older people and general surgery) of the same type as the study site. Maternity was excluded as pressure ulcers and falls were not applicable and infection rates low. Data sources were discharge data from the patient administration system (including patient age and diagnosis) and incident reporting system for safety surveillance and infection control purposes (see online supplementary appendix). Time series were analysed by means of statistical process control charts (SPC, u-charts) to look for evidence of special cause variation associated with the move to single rooms.^36^ Where special cause variation was found, we further explored data to assess whether it could be attributed to the move to 100% single rooms. To attribute a change to single rooms, we explored change in case mix as an alternative explanation and looked for a similar but attenuated pattern in the new-build control where the proportion of single rooms increased. For space reasons, we report safety or infection events (eg, per falls or medications errors) per 1000 patient-days in four phases: ≥10 months before moving, 1–9 months before moving, 1–9 months after moving and ≥10 months after moving.

### Costs associated with single rooms

We conducted a comparative analysis of costs associated with single rooms. To do this, we collected data on bed occupancy, administrative data on cleaning costs, nurse staffing, payroll, length of stay and build costs. Structured observation data were also analysed for proportion of time spent on direct care with patients. We modelled the impact of these costs over the lifetime of the hospital in discounted cash flow/net present value terms. In addition, 12 experts from the architecture, construction, hospital and facilities management and operations were interviewed to inform analysis on the relative impact of different hospital designs on costs and resource use (see online supplementary appendix).

## Results

### Patient experiences

At interview, patients in the shared accommodation wards reported enjoying the security of being very visible to staff and to each other, which resulted in good patient camaraderie for some. However, the disadvantages of shared patient accommodation included exposure to confused and disruptive patients, lack of privacy and lack of physical comfort. Four dimensions reflect patients’ overall experiences of shared patient accommodation: security, community (advantages and disadvantages), lack of privacy and lack of physical comfort. Four dimensions reflected patients’ overall experience of single-room wards: comfort, control, connection and isolation (table 1).

**Table 1:** Patient advantages and disadvantages of shared patient accommodation and single-room accommodation

*Shared patient accommodation in an open ward*	
Advantages	Patient interview data
**1. Security—visibility and staff proximity**	
Shared patient accommodation meant nurses’ station visible from patient beds: (i) Enabled patients to see staff and evaluate staff competence, leading to a sense of safety (ii) Staff were close by at all times, and this proximity also increased patients’ perceptions of security (iii) Shared patient accommodation allowed patients to witness the care of others which helped patients feel they were in a safe environment	*“You were so close to the station anyway so if something did go wrong you could call somebody. They didn't draw the curtains so they could see you all night…they did keep a good watch on you”* Patient, MAU (female—age 55) “*You could always see that there was a nurse there….You were never ever left alone…and to me that's important because if you press the buzzer, they look up, they're with you within seconds….”* Patient, surgical ward (male—age 73)
**2. Community—patient camaraderie**	
(i) Proximity of other patients created a ‘ready-made’ community which many patients appreciated (ii) Watching or observing other patients passed the time or distracted patients from their own discomfort (iii) Actively interacting with other patients to provide and obtain emotional support was noted by many as important.	*“In the bed I was in, you had a nice little community of us, all within talking distance…It's good for morale…[it] created a wonderful atmosphere between…we got on well with one another. And we all knew what each other had got wrong with them.”* Patient, surgical ward (male—age 73)

**Disadvantages**	**Patient interview data**

**3. Community—confused /disruptive patients**	
'Community’ dynamic could change quickly; positive aspects negated: (i) When another patient became confused, or when a disruptive patient was admitted to the ward (ii) Patients described feeling vulnerable and concerned, for the patient's welfare and sometimes their own (iii) Staff were ‘tied up’ caring for or dealing with an individual patient—leading to a reduced sense of security/emotional comfort	*“The man who was opposite…he'd got dementia and he was in the shouting stage…shouting out ‘Help’ all the time…they tried everything to help him; they were terribly, terribly patient… most of us, well I think all of us, understood, but it was a nuisance, because in the end it took over, you couldn't do anything else but listen, and see how they were getting on with him.”* Patient, surgical ward (male—age 71)
**4. Lack of privacy—confidentiality and embarrassment**	
(i) Some patients accepted a lack of privacy as an unavoidable aspect of the environment (ii) Others were left feeling vulnerable, particularly on the maternity ward	*“It's difficult to keep everybody private…..they'd pull the curtains but you obviously can hear what they're saying… if you're relatively well I think it might be a factor but once you're so ill you really don't care about anything.”* Patient, MAU (female—age 55) “*I had patients with families peeking through the curtains that didn't fit properly. It was awful. I was so embarrassed…There's no privacy”* Patient maternity ward (primiparous) “*I was trying to keep him [baby] quiet to try not to disturb everybody else….I was getting really anxious because I was thinking, ‘Oh, don't wake everybody else up.’”* Patient, maternity ward (primiparous)
**5. Lack of physical comfort—noisy and no control**	
(i) Aspects of physical comfort of importance to patients included location and size of shared toilet facilities, lack of space around the bed, and inability to control lighting, temperature and noise	*“I had two drains to carry with me and your drip thing to get to the bathroom, and it pulls all your nightie up at the side ….You're constantly looking somewhere else, trying not to make eye contact because I get very embarrassed.”* Patient, surgical ward (female—age 57) “*I got no sleep because it is just so noisy…..the other babies screaming…. So it was very difficult to sleep in a maternity ward like that.”* Patient, maternity ward (primiparous)

*100% single rooms*	
Advantages	Patient interview data
**1. Comfort—en-suite convenience, rest and sleep-‘hotel’** Patients experienced high levels of comfort in the single rooms, in relation to: (i) En-suite toilet facilities, lighting, ventilation, having a view from a window and noise levels (ii) The single room was frequently compared favourably with a hotel or home environment (iii) The design of the room helped reduce patients’ anxiety, promote rest and sleep, and support physical comfort	*"You had a shower, toilet, and washing facilities. Got your own TV […] I would rate it really as probably on a par with a three star hotel […] That's how good it was.”* Patient, surgical ward (male—age 70). “*I only just made it into my bathroom in time […] I would have been so embarrassed, if I'd had to go out of my [room] and across the corridor and feel sick, and wet myself, I would just die of embarrassment […] but there it was absolutely fine.”* Patient, surgical ward (female—age 45) “*The single room is, I think, the most perfect solution they came up with because […] sometimes babies are crying constantly, […] at least if baby was asleep, I could also go to sleep because there wasn't anyone next to me with a baby crying or anything.”* (Postnatal patient, age 26)
**2. Control—enhanced privacy and freedom- ‘at home’** (i) Control rarely featured in patients’ accounts of staying on shared patient accommodation (ii) Control was closely associated with privacy and freedom; not being observed by others; retaining independence and agency- patients valued not having to worry about what other patients might be thinking about them (iii) Patients compared the single room with being at home, in their own space, with similar control and privacy (iv) Privacy and flexibility of the single-room environment for receiving visitors	*"Being in a room on my own where I didn't have to talk to people if I didn't want to, I could watch the telly […] And the fact that I'd got privacy, and things like being able to go to sleep when I wanted, and wake up when I wanted, was really beneficial […].”* Patient, MAU (female—age 44) “*It just gives you time to properly relax, and you haven't got other people shouting across the ward… It's just nice because you can relax in your own time. You're given that privacy, and that's what I like.”* Patient, surgical ward (female—age 72) “*You can ask them anything […] I had a C-section and I would ask them about the pains, the bleeding, the catheters… Because it's private, I felt comfortable and they could even show me and […] actually demonstrate it for me*”. (Postnatal patient, age 26) “*I do think visiting in here is very much easier, in single rooms. [There's] more space. And there's more confidentiality, you feel not everyone's hearing your business […] it is nice really [visitors] can come whenever they like and that's a very big bonus.”* Patient, old people ward (female—age 80)

**Disadvantages**	**Patient interview data**

**3. Connection—seeing staff and being seen** (i) Anticipating the move to single rooms patients in shared patient accommodation expressed anxiety about staff not being in close proximity and able to monitor them sufficiently (ii) Proximity and monitoring not directly mentioned by patients in single-room accommodation who spoke more generally about feeling or not feeling alone (iii) The quality of interaction with staff experienced by patients in single rooms affected how connected they felt to staff (iv) Some patients thought that the single room enabled staff to give them undivided attention and supported one-to-one care, others that staff were ‘*very distant*’, ‘*busy*’ and ‘*did not have time to chat*’ (v) Not being able to see staff going about their work beyond the single room meant patients often had little idea of other patients needs or how busy or not busy staff were	Anticipating the move a patient in shared patient accommodation said: “*The problem is if you have these low blood pressure episodes they happen so quickly that you might not have a chance to ring the bell. And that would have worried me. Say you'd got up to go to the bathroom and then had one of these episodes and fallen over…then you just do wonder how often they would come along and check on you….I would have thought that's not a good idea.”* Patient, MAU (female—age 55) “*They didn't really have time to chat […] They just came in and did what they had to do, the temperatures and blood pressures, and things […] You get more time [in a multi-bed bay], not to chat to the staff, but to ask them questions, because they're in a bay more than they are in a single room. They would only come to your room just to see if you're okay, every so often or if you called them.”* Patient, surgical ward (male—age 71) “*If you're in hospital, you're ill, you're not there for a holiday, and if you're ill you need a lot of treatment, you have your blood pressure taken three times a day, your temperature …So you are being constantly attended by nurses because of your condition….. so actually, a [single room] is a very busy place, you're not on your own, hardly ever.”* Patient, surgical ward (male—age 84)
**4. Isolation** (i) Anticipating the move to single rooms patients in shared patient accommodation expressed anxiety about potential isolation in single rooms (ii) For some patients in single rooms, lack of interaction with other patients led to strong feelings of isolation (iii) Patients who felt little connection with staff were likely to report feeling isolated in single rooms (iv) Some patients wanted to have the opportunity to socialise with other patients to counter this isolation (v) Patients who missed ‘company’ felt confined in their rooms and unaware of dayroom opportunities or whether it was permissible to walk the corridors or ‘visit’ other patients	Anticipating the move a patient in shared patient accommodation said: “*I think…single bed [rooms] are a bit isolating. You wouldn't see life going by. Yes, it would be quieter, but perhaps a bit too quiet? [other patients] they're not going to go into an individual room really to say ‘Hello’ and see how you're getting on, rather than if they're just passing by the bed, will they?”* Patient, surgical ward (female—age 56) “*It would have been nice to have been able to see other people, or maybe just to chat with other mothers […] If you're in an open ward, you just see people coming and going […] you can chat to people across the ward […] I just wanted to get home […] because there isn't anyone to chat to and I just felt a little bit isolated being in the room on my own.”* (Postnatal patient, age 36) “*It did get a little bit lonely […] you don't really get to talk to many people, other patients. […] I wanted to go for a walk just to get out of the room and maybe have a chat with someone. You do start to feel a little bit stir crazy stuck in a room […] I kind of felt like I had to stay in my room.”* Patient, MAU (male—age 44)

Patients experienced high levels of comfort in the single rooms and compared the hospital room favourably to a hotel or home environment allowing them to experience the comfort and control of home or a hotel, in hospital. Patients also experienced a high degree of personal control in the single room; feeling not as ‘on show’ as in shared patient accommodation. Patients in single rooms described being able to do as they pleased at any time without worrying about other patients. This was in contrast to patients’ experiences in shared accommodation.

Patients appreciated the confidentiality afforded by the single room, and the privacy and flexibility it gave for visitors. Patients rarely used dayrooms (communal social spaces) (available on two wards); they were only orientated to their room. They did not know where dayrooms were and were uncertain if they could leave their rooms. Interaction with other patients was largely absent in the new hospital, leading to a sense of isolation for some patients. Patients reported regular visits to their single room by various staff, and all patients described nursing staff conducting frequent intentional ward rounds. These nursing rounds were introduced as a method of ensuring all patients were observed and had their needs met regularly and to counter isolation in single rooms. Some patients experienced a good quality of communication and felt connected with staff; others experienced care as largely task-driven and functional, which may have enhanced the disconnection experienced by some patients.

Overall, the majority of patients (two-thirds of those interviewed) expressed a clear preference for single rooms. Privacy and en-suite bathrooms were important aspects of this. All the women in the postnatal unit liked single rooms. One-fifth of patients, including almost half the men interviewed, said they preferred multi-bedded accommodation. Staff associated loneliness with older patients, but it was experienced by patients in all age groups in this study. Single rooms were a clear preference for many patients, but a significant minority, including patients of varying ages and patient groups, wanted greater choice, either in terms of having the option of shared accommodation or being able to interact with other patients in communal spaces of the hospital. Approximately one in three patients interviewed (11 of 32) reported lack of interaction with other patients as a main disadvantage of the single room.

### Staff experiences

At interview, before the move to 100% single-room hospital, staff identified the advantages of shared patient accommodation as good visibility of all patients, which facilitated surveillance and monitoring; supporting good teamwork, which was enhanced by being able to see and find staff easily that enabled staff to support each other. Staff also identified that shared patient accommodation allowed social contact between patients, which helped staff and enhanced recovery (see table 2). Disadvantages of the shared patient accommodation included an inadequate physical environment for patients and for care delivery and poor privacy, sleep and rest for patients.

**Table 2:** Staff advantages and disadvantages of shared patient accommodation and single-room accommodation

*Shared patient accommodation in an open ward*	
Advantages	Staff interview data
**1. Visibility—enhanced surveillance and monitoring**	
Visual and aural proximity of staff to patients resulted in three key perceived benefits for staff: (i) Enhanced surveillance and monitoring of patients (ii) Staff ability to monitor and communicate with patients enhanced by the proximity of patients to each other (iii) Staff benefitted from patients acting as an extra pair of eyes on the ward	*“Even when I'm sitting here by the desk [staff base], I can see…By just looking up, you can automatically see that, ‘Oh, I think he's not breathing well,’ or something is going wrong, and you can act immediately.”* E6 Nurse, medical (older people) ward “*I was on [the postnatal section] the other night in the early hours of the morning and I was seeing somebody and I could hear this baby throwing up, so I dived behind the curtain so I could get it on its side, pat on the back. So, yeah, you can see the patients as you walk down very easily. You hear things.”* M3 Midwife, maternity ward “*Where I am today it's been absolutely hectic because we've got confused patients. They're all constantly shouting out. And where we are now, we can shout over, ‘I'll be with you in a minute.’ [It helps with] knowing where to go, how to prioritise yourself really”* A1 Healthcare assistant, MAU “*We've got patients who look out for [other patients]. If a patient next to them sees that they're having trouble doing something they'll ring their bell for them and say, ‘Oh, nurse, she's doing this, she's doing that, I think she's trying to stand up…’ which is really good.”* E3 Healthcare assistant, medical (older people) ward
**2. Teamwork and communication**	
(i) Staff valued being easily able to request or provide assistance where needed (ii) In staff survey, surgical and medical (older people) ward staff had the highest mean scores for the items ‘Obtaining advice from colleagues’, ‘Finding a staff member’ and ‘Knowing when other staff might need a helping hand’ (mean scores >4) (iii) Staff learning from each other and supporting each other highlighted as important. During observation sessions ward managers were frequently observed on the ward ‘floor’, assisting and encouraging or advising their team	*“It's one of the lovely things about the Nightingale wards, at least we [nursing staff] can all see each other… you can go, ‘Do you want some help there?’* E2 Healthcare assistant, medical (older people) ward “*Sometimes you are faced with a situation that is difficult to manage on your own and so there's nearly always somebody very close by that you can call. Or if maybe you were struggling a bit in any way …somebody else would probably hear and come to your rescue. Those are big advantages.”* S3 Nurse, surgical ward
**3. Facilitation of social contact between patients**	
(i) Staff were positive about patients being able to see, hear and interact with each other in shared open plan areas (ii) Social contact between patients was seen as an aid to staff on busy wards, who might not have time to interact for longer periods with patients (iii) Social contact was perceived to support recovery and relieve boredom on the wards	*“If you have one end of the ward where everybody's quite jolly, it really lifts the spirits of everybody. And it's very distracting. If you're sick in a room on your own and it's quiet and all you've got to think about is your pain, whereas if you've got distractions of people walking around, talking to you, it's a really good therapy.”* S6 Nurse, surgical ward “*Other patients will give somebody a boost, and they will talk to them [another patient] and they'll say, ‘Oh come on, try and eat a bit more’…that helps people a great deal, if somebody's there talking to them…If we've got two people who are reasonably well and they like a chat…we do try and juggle the beds around so that they're together, so that they can have a talk.”* E2 Healthcare assistant, medical (older people) ward

*Shared patient accommodation in an open ward*	
Disadvantages	Staff interview data
**4. Inadequate physical space for patients/care**	
(i) Lack of space for staff to provide nursing care to patients emerged as a key constraint (ii) Lack of space in toilets and showers made it difficult for staff to assist patients and created obstacles for patients attempting to mobilise independently (iii) Problems created by lack of space included risk of musculoskeletal injuries, trip hazards and a perceived increase in time required to undertake tasks	*“You're banging into the next patient's chair or locker and stuff like that. It's really tight around the bed space, and .. when trying to get a commode in there you're fighting with everything really.”* S4 Nurse, surgical ward* When you want to hoist someone…it takes so much time, you've got to move the bed that side, you've got to move the [other furniture], you know. It's so, so much energy doing that, rather than if there was enough space, you just wheel it in, hoist your patient, take them away, and that's it.”* E6 Nurse, medical (older people) ward
**5. Poor physical spaces/facilities for staff**	
(i) Space at staff bases generally considered inadequate—limited space and poor access to the limited number of PCs and telephones (ii) Staff break rooms had limited facilities and no kitchen. Often dark, cold and shabby, and multipurpose, serving as meeting rooms as well as staff break rooms (iii) Lack of staff toilets was problematic (particularly maternity and surgical wards), meaning staff often had to go away and return later to use facilities	*“You often have nurses sitting there, then you'll get physios and OT coming along, they might want to sit down and there's only one screen [PC] in the ward and that's really not enough, we need more screens to be effective because you're waiting to use the screen and so it would be useful to have more computer stations.”* S1 Nurse, surgical ward “*There are no sofas in it [staff room], as you can see [ ] people's bags and coats, and it's usually like a stock room…There's normally stuff piled…It's normally quite cold in here as well, it's got the old window.”* M5 Student midwife, maternity ward “*Toilet facilities are horrendous, one toilet for all of us, doctors, nurses, domestics—horrendous. So many times you walk up and there's somebody in there. ..[The] changing facilities…[are] totally inadequate, totally.… there's nowhere to change.”* S6 Nurse, surgical ward
**6. Poor privacy, sleep and rest for patients**	
(i) Staff were looking forward to improved privacy, sleep and rest for patients (ii) Patients in the old accommodation were disturbed, for example, by general ward activity, or by other patients, which could create anxiety	*“On [the antenatal area], because you're doing sort of CTGs the whole time [ ] you can always hear like other peoples’ babies’ heartbeats going on… it's so noisy on there, whatever happens you can hear. Like the other day…someone delivered on the ward [because there wasn't enough time to get her to the Delivery Suite] and…The other women on the ward they were just sort of looking as if to say, ‘Is this really what it's going to be like?’ and so in that sense it's not good.”* M5 Student midwife, maternity ward

*100% single rooms*	
Advantages	Staff interview data
**1. Privacy, dignity and confidentiality: more personalised patient care**	
Staff compared single rooms favourably with open-plan wards in relation to: (i) Patients having their ‘*own space*’, including an en-suite bathroom and toilet, where personal care took place away from other patients (ii) Allowing patients to sleep, rest and recuperate without disturbance (iii) They could also better accommodate visitors. (iv) Privacy was particularly valued for patients who were seriously or terminally ill and for their families (v) Staff also reported less likelihood of interruption and distraction	*“*[I would] *hate to go back to nursing patients behind curtains*” because “*dealing with gastric surgery, it can be embarrassing when patients don't make it to the toilet in time […] now they have the dignity and privacy of being in their own room.”* S16 Nurse, surgical ward “*I think from a privacy and dignity point of view, when patients are dying, or when relatives are in there, or in extremis, when you don't really want the next patient to be making a noise, then [single rooms are] fantastic. […]”* KSI28—consultant “ *… [at the old hospital] you'd have to find a room outside the ward to bring the family to, to have a discussion with them, whereas here, you might be able to speak a bit more openly and privately with the family and with the patient present. Almost have a mini MDT with the family and the patient in their room.”* AHP40—dietician “*You're able to give your patient the attention and care because you're with them; you haven't got someone else shouting from the other side of the room for you.”* M01-Healthcare assistant, MAU
**2. Improved room design: improved care delivery**	
(i) Improved working environment for delivering patient care and rooms described as modern, clean, spacious and safe (ii) The en-suite facilities one of the most positive aspects, allowing nursing staff to more easily assist patients with personal care	*"[The single rooms] are really accessible and for patients that want to hold on to things while they're walking, they've got bars around the side, from the bed to the bathroom, they've got the bars alongside of the toilet and the bars on the wall to hold onto, bars by the shower, so I think that's really good. The floors are non-slip as well, so I stand patients up quite confidently in there, without worrying that they're going to slip.”* S14—Healthcare assistant, surgical ward
**3. Improved ward layout and design**	
(i) Centralised ward support facilities (eg, clean and dirty utility, drug preparation room, kitchen) liked by staff (ii) Secure ward entry system to allow patients and visitors to enter and exit (see also disadvantages) (iii) Dedicated staff break rooms (on surgical ward and the older people ward)	"*The clinical room is a good size, much bigger than at the old hospital […] we've got lots of surface area, whereas before it used to get a little bit cramped, and we'd be getting confused and our drug charts muddled up. Now I think there's much more space to do things quickly and more effectively.* S16—Nurse, surgical ward “*We've got up to 30 patients, possibly confused, wandering; unless a member of the staff has opened the door, those patients are safe […] that's priceless really.”* OP25—junior sister, older people ward “*It's much cleaner, much tidier, somewhere to sit down and eat […] you've got access to fresh air which we didn't really have at the old place, so I think the staff areas are much nicer.”* S14—Healthcare assistant, surgical ward
**4. Perceived reduced risk of infection**	
(i) Staff anticipated that single rooms would reduce infection risk (ii) New wards were perceived by staff to support good hygiene practices, with no-touch clinical hand washbasins in every patient room (iii) Personal protective equipment and alcohol hand gel and antibacterial wipes were available from wall-mounted dispensers outside every room. This provided a visual reminder for staff entering a patient room to comply with infection control procedures	*"I've taken a picture of the gloves on the wall, just outside the general patients’ room, the gloves, alcohol dispenser, aprons, and the wipes […] they're very clean, clear, easy to refill and they really benefit from being where they are, which is perfect, the fact that they're outside every single patient's room, there's no question about it.”* OP25—junior sister, older people ward “*Because you've got individual basins, you're not sharing a sink. You're able to wash your hands very quickly after having patient contact, rather than having to walk around looking for a basin to use. […] So you just feel that you're able to carry out infection control procedures more effectively.”* S16—Nurse, surgical ward

*100% single rooms*	
Disadvantages	Staff interview data
**5. Reduced visibility: difficulties monitoring and safeguarding patients**	
(i) Staff described visibility of patients from the ward corridor as limited to the patient room they were directly outside, and then only if the door or vision panel in it was open (ii) The line of sight into the patient room was interrupted by the wall of the adjacent room's en-suite bathroom (‘in-board’ single-room design) also obscuring the view of patient room doors for staff looking down the corridor (iii) All staff interviewed perceived that lack of visibility of patients in the single-room wards had contributed to an increase in falls in the new hospital (iv) Experienced nursing staff thought that it had been easier to prevent falls in multi-bed accommodation because they could ‘keep an eye’ on patients and were more aware of warning signals, for example, patients attempting to get up from their chair or bed (v) Staff adapted work patterns to monitor and safeguard patients, but use of cordless telephones to locate each other was variable	“*When we were on an open ward I could walk on the ward and I could view everybody. And when you knew your patient you could see [if they didn't look well]. Whereas now I can walk up and down the rooms, but as soon as I'm in a room I'm away from everybody. […] That time has gone where you could just stand and have a quick chat with a patient while you were still keeping an eye on everybody else.”* S11—Nurse, surgical ward “*Last week, we had about three people […] climbing out of beds and falling […] And I know on an open ward [patients] can still climb out of bed but at least as you're walking up and down the ward you could physically see them […] now the only time we know somebody has fallen out is when we hear the clump and they're on the floor.”* S15—Nurse, surgical ward “*You have to adjust your nursing practice just to make sure that everybody is seen and you keep an eye on them the whole time. With the bays you'd go in to see one patient but then subconsciously you're eyeballing everybody else, making sure everybody else is okay. Whereas here you have to physically go into each and everybody's rooms, or stop and have a look at them.”* M03—Nurse, MAU “*I walk around with my head permanently fixed to the side that all the rooms are on, just checking […] I've just adapted. I now look into every single room every time I walk past and I make the effort to go into the rooms.”* M05—junior sister, MAU
**6. Social isolation of patients**	
(i) Staff felt social isolation was a disadvantage of single-room wards compounded by ward design with limited day rooms (ii) Social isolation influenced, patients’ satisfaction with their hospital stay and their emotional well-being and recovery (iii) Staff felt that provision of social or communal space for patients should have been given much higher priority (iv) Staff felt older patients likely to be more lonely (though our patient data does not support this from our sample) although staff also recognised that patients of all ages could be disadvantaged by not mixing with others and hearing their experiences, which was thought to help individuals assess their own progress and could be reassuring, motivating and encouraging (v) Socially isolated patients were seen as likely to make more demands on staff, for example pressing call bells frequently or talking a lot to ‘keep’ nurses in the room with them which created tensions for staff	“*I just do sometimes feel sorry for the older patients that are in for weeks [and] don't necessarily have a lot of contact with other people. And I guess mood has a massive impact on everything in hospital, on your recovery, on your eating, on how likely you are to get up and work with the physio that day. And sometimes if you've other patients motivating you, or even just speaking to you, it just picks up your mood, it can help.”* AHP40—dietician “*For postnatal ladies it's important that they can see what's going on around them and it's not all about them and their baby […] they don't understand that every baby feeds all the time and cries and everything. They can't see what is normal and I think they think, ‘My baby is going to feed and go to sleep for six hours.’ But if they see the other women struggling as well it sort of normalises it for them.”* PN36—midwife, postnatal ward “*It tends to be older patients who have no company at home […] especially if they're being barrier nursed and the door needs to be closed, they find it very isolating, and I've had quite a few older people get quite upset. And then the impact it has on us because they're lonely, they'll be pressing their bell all the time for nothing other than just wanting someone to be there with them, [but] you just don't have that time […] Yeah, and if they don't see you for a little while, patients often think you must not be doing anything.”* M01—Healthcare assistant, MAU
**7. Maintaining teamwork and communication**	
(i) After the move, staff felt that the quality of teamwork had been difficult to recreate (ii) Nurses described seeing less of their colleagues, being unaware of what was happening in other parts of the ward, and sometimes feeling isolated (iii) One of the main difficulties described by staff was finding colleagues to obtain assistance and information. This was a cause of much frustration, especially for HCAs, use of technology to support this was variable (cordless telephones) (iv) Nursing staff worked in small, decentralised teams, caring for patients in a cluster of eight to ten rooms, rather than one large team. Some staff liked this, but others felt that the mutual support they had experienced on multi-bed wards could not always be relied on in the new hospital (v) Single-room wards impacted on the ability to support, train and develop staff as more difficult to supervise junior staff (vi) There were also fewer opportunities for informal learning than on multi-bed wards	*"It can be a bit difficult sometimes, if you're really stuck and, you know, I've been hanging out of rooms calling for a nurse sometimes, but if somebody's in another room, you can't see them. […] [If] you really need a nurse, or you really need somebody to come and help you, then you have to go through all the rooms to try and find them […] So if you do need assistance […] we often just press the call buzzers ourselves.”* S14—Healthcare assistant, surgical ward “*Well, I suppose sometimes, on a really busy day, you can feel a bit isolated […] I know you shouldn't, but at times it does make you put your own health at risk. I've done that with my own back, you just think, ‘Oh I can't find anyone, [the patient] desperately needs the toilet, I'm going to help them.’”* OP23—Healthcare assistant, older people ward *”There's a feeling that it's almost like three separate wards, in a way. You're very much self-contained within your own team. Whereas before, I think there was far more interaction between nurses.”* S16—staff nurse, surgical ward “*That is a direct result of the environment really, because you can't eyeball nurses enough to know that they're drowning and they need help.”* M06—Nurse, MAU “*You overhear someone working behind a curtain and you pick up and you think, ‘That was a really nice thing they did for that patient. Maybe I'll try that.’ I think that's definitely missing, picking up on things from each other that way, because […] it's not as easy to hear how they interact with people.”* M05—junior sister, MAU

MDT, multidisciplinary team.

Following the move, staff reported that single rooms improved privacy, dignity and confidentiality for patients and were better for visitors. Single rooms were perceived to facilitate communication with patients, with reduced interruptions and allowed more personalised care. The single rooms provided an improved working environment for care delivery, being modern, clean and spacious, and the en-suite bathrooms made personal care easy to deliver. Staff also noted the improved ward layout and felt the single-room environment reduced the risk of infection (table 2).

Analysis of pre-move and post-move survey data found staff perceived statistically significant improvements in the efficiency of the physical environment, the patient amenity, the effect of the environment on infection control, patient privacy, and family and visitors. The largest increases were found for perceptions of infection control and patient privacy (see table 3). The new wards were perceived by staff to support good hygiene practices; however, there was a perception by infection control staff that single rooms induced a degree of complacency in relation to basic precautions and there was a perceived need to reinforce infection control procedures and protocols. Basic infection control principles were revisited to counter the belief that single rooms protected from hospital-acquired infections. Perceptions of the effect of the single-room environment on teamwork and staff's ability to deliver high-quality care were significantly more negative after the move than before (see table 3).

**Table 3:** Results of t tests comparing staff perceptions of the ward environment pre and post move

Scale	Phase	Mean†	SD	t	p-Value
Efficiency of physical environment	Pre	2.81	0.67	−3.346	0.001*
	Post	3.24	0.70		
Ability to deliver high-quality care	Pre	3.20	0.66	2.59	0.011*
	Post	2.88	0.67		
Staff amenity	Pre	2.95	0.67	−0.373	0.710
	Post	2.99	0.73		
Patient amenity	Pre	2.82	0.64	−5.52	<0.001*
	Post	3.43	0.50		
Layout supports infection control	Pre	2.75	1.11	−8.39	<0.001*
	Post	4.25	0.73		
Patient privacy and confidentiality	Pre	2.54	1.15	−10.14	<0.001*
	Post	4.31	0.59		
Teamwork and training	Pre	3.55	0.64	4.34	<0.001*
	Post	2.96	0.77		
Patient safety	Pre	3.23	0.69	−7.32	0.466
	Post	3.32	0.67		
Staff safety	Pre	3.19	0.68	0.67	0.502
	Post	3.10	0.75		
Interaction with family/visitors	Pre	2.74	0.77	−8.60	<0.001*
	Post	3.90	0.63		

*p<0.05. †Higher scores on the scales indicate more positive responses than lower scores.

Staff rated the post-move single-room accommodation as significantly lower for their ability to deliver high-quality care (table 3). A notable disadvantage that staff highlighted at interview was the reduced visibility offered by the single-room design, making patient surveillance difficult. Staff identified various obstacles to safe and efficient working, one unique to this particular hospital design (*in-board* bathrooms that protrude into corridors obscuring views into more than one room at a time). Staff saw their greatest challenge as monitoring and keeping patients safe, especially those at risk of falls. Although staff found the initial increased rate of falls distressing, their rating of patient safety in the survey was not significantly different from pre move (table 3). All staff interviewed perceived a lack of visibility of patients in the single-room wards had contributed to an initial increase in falls in the new hospital. Experienced nursing staff felt it easier to prevent falls in shared patient accommodation because they could ‘keep an eye’ on patients and were more aware of warning signals, for example, patients attempting to get up from their chair or bed. Nurses had to work differently and adapt their working practices to ensure all patients were seen regularly, requiring teamwork with colleagues and the initiation of regular hourly intentional rounds. Other successful examples of new ways of working included supervisory roles for situation awareness; falls interventions and starting a lunch club in a shared social space for patients on the older people's ward to combat isolation and improve food intake at lunchtimes. Nurses found time management and prioritisation of workloads challenging in the new environment and struggled to divide their attention between all the patients they were caring for (they had found this easier in shared patient accommodation). Staff recognised that they required different strategies to enable them to divide their time between patients and feel satisfied that they were giving all patients sufficient personalised care (again easier on multi-bedded wards), but this challenge had largely been left to individuals to resolve, with reportedly limited success and associated dissatisfaction (table 2). Trial and error was a feature of innovations, with staff teams trying, for example, different ways of preventing falls and different configurations of decentralised teams.

Social isolation was perceived by staff to be a real disadvantage for both staff and patients. Staff also felt that the quality of teamwork they had enjoyed on shared accommodation wards was difficult to recreate. Nurses described seeing less of their colleagues, being unaware of what was happening in other parts of the ward and sometimes feeling isolated. Patients were nursed in three groups of 10 patients by sub-teams of nursing staff. Staff adapted to these decentralised nursing teams, but information exchange within wider nursing teams was perceived to be worse. Locating colleagues to obtain assistance was one of the main difficulties described by staff and was largely unresolved by new ways of working and by use of communication technology (table 2).

Pedometer data showed an increase in the mean number of steps per hour for all wards and staff groups (table 4).

**Table 4:** Mean steps per hour by wards, pre/post move and staff group

	Pre-new build				Post-new build			
	No. of observation sessions	Mean	Range	SD	No. of observation sessions	Mean	Range	SD
Ward								
Maternity	14	630	380–1007	194	8	687	463–1008	211
Surgical	17	653	354–996	152	22	793	419–1274	247
Older people	23	664	361–965	158	21	845	553–1229	193
MAU	19	773	479–1007	181	16	880	469–1311	254
Staff group								
Midwife	7	475	380–640	94	6	583	463–683	99
RN	32	639	354–1007	178	29	827	419–1311	240
HCA	34	768	470–1007	131	32	853	469–1274	220
Total	73	683	354–1007	175	67	817	419–1311	231

HCA, healthcare assistant; RN, registered nurse.

The modelling, which adjusted for ward, staff group and day of week, showed a significant increase from a mean of 716 (95% CI 660 to 771) before the move to a mean of 839 (95% CI 787 to 892) after the move (F(1, 83)=10.36, p=0.002). HCAs (mean 836, 95% CI 776 to 895) walked significantly further than RNs (mean 719, 95% CI 667 to 772, F(1,83)=8.01, p=0.006), but there was no significant difference between wards in the distance walked (F(2, 89)=2.38, p=0.099). The effect of the move was not moderated by either ward (F(2,82)=0.64, p=0.53) or staff group (F(1,76)=3.48, p=0.066).

Analysis of the time and motion observation data showed an increase in the proportion of direct care, indirect care, professional communication and medication tasks and a decrease in ward-related activities such as cleaning and bed-making, but none of these were statistically significant. There were, however, fewer interruptions during tasks and work was less fragmented. There were no significant differences in staff well-being and stress post move.

**Figure 1:**
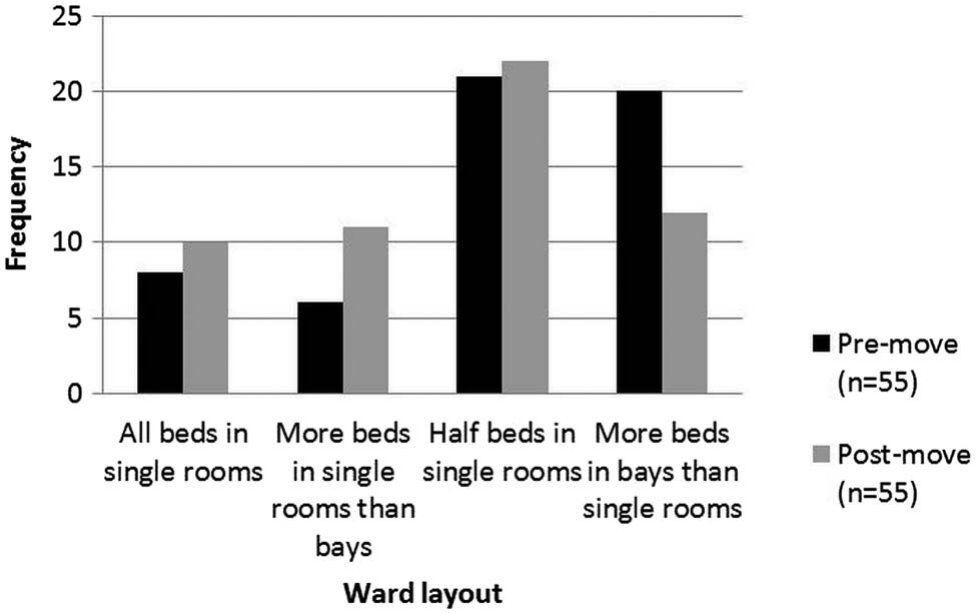
Nurse preferences for single-room or multi-bed accommodation.

Overall, when asked their preference, most staff said they would prefer wards with a mix of single rooms and multi-bedded accommodation both before and after the move. After the move, only 18% (n=10) of staff indicated a preference for 100% single rooms (figure 1). The most common preference, before (38% n=21) and after (40% n=22) the move, was a combination of 50% of beds in single rooms and 50% in bays. More staff in the pre-move survey reported a preference for more beds in bays (36%, n=20) than in the post-move phase (22%, n=12).

### Impact on patient safety

There were few changes in safety outcomes that might be attributed to the move to single rooms. Only 5 of 15 SPC charts across the 5 outcomes from 3 wards at the single-room hospital showed special cause variation following the move (bolded in table 5). Increases of falls and medication errors in the MAU immediately following the move were found but rates decreased to previous levels 9 months after the move. No equivalent change was observed in the ‘new-build’ control hospital. Increases in *C. difficile* infection, falls and pressure ulcers post move were observed in the older people's ward. Again, no equivalent change in outcomes was observed in the new-build control hospital. However, a reduction of about 50% in length of stay (LOS) indicates a substantial change in case mix on the older people’s ward and so these outcomes could not clearly be attributed to the move or the environment.

**Table 5:** Safety and infection control outcomes before and after the move

		Before		After	
		1–11 months	12–20 months	21–29 months	30–36 months
*100% single room*					
MAU					
LOS	Per 1.000 patient-days	1.21	1.03	1.49	1.3
Falls		3.9 (2.5 to 6.1)	2.3 (1.2 to 4.4)	**13.9** (**11.3 to 17)**	**0.7** (**0.2 to 1.9)**
Med. errors		1.3 (0.6 to 2.8)	0.5 (0.1 to 1.9)	**8.2** (**6.2 to 10.6)**	**0.7** (**0.2 to 1.9)**
Older people's ward					
LOS		36.17	40.14	20.77	19.01
Falls		2.8 (2.1 to 3.9)	2.4 (1.6 to 3.6)	**9.5 (7.8 to 11.7)**	13.3 (11 to 16.2)
Pressure ulcers		0.4 (0.2 to 0.9)	0.2 (0 to 0.7)	**3.3 (2.3 to 4.7)**	3.5 (2.4 to 5.2)
*Clostridium difficile*		0.1 (0 to 0.4)	0.1 (0 to 0.4)	0.2 (0 to 0.8)	0.5 (0.2 to 1.4)
*Control new-build hospital*					
MAU					
LOS		0.78	0.91	0.95	0.98
Falls		4.1 (1.3 to 11.1)	2.1 (0.5 to 6.6)	7.0 (3.9 to 12.2)	7.5 (2.8 to 18.4)
Med. errors		4.6 (2.4 to 8.4)	11.8 (7.1 to 19.2)	7.0 (3.9 to 12.2)	0.1 (0 to 7.1)
Older people's ward					
LOS		8.16	8.36	7.53	7.86
Falls		6.9 (4.6 to 10.4)	9.7 (7.3 to 12.7)	11.2 (9.3 to 13.5)	12.6 (9.1 to 17.4)
Pressure ulcers		4.0 (2.7 to 5.9)	3.6 (2.2 to 5.6)	1.4 (0.8 to 2.4)	2.9 (1.4 to 5.7)
*Clostridium difficile*		1.6 (0.9 to 2.8)	1.1 (0.4 to 2.5)	0.3 (0.1 to 0.9)	0.3 (0 to 2.1)
*Steady-state control hospital*					
MAU					
LOS		6.88	6.37	5.96	5.42
Falls		23.8 (21.2 to 26.7)	**14.6 (12.4 to 17.2)**	17.8 (15.2 to 20.8)	20.2 (15.5 to 26.3)
Med. errors		N/A	N/A	N/A	N/A
Older people's ward					
LOS		21.98	36.59	35.88	45.64
Falls		29.7 (25.9 to 34)	**17.4 (14 to 21.5)**	12.1 (9.7 to 15.1)	5.9 (3.4 to 10.1)
Pressure ulcers		1.4 (0.7 to 2.7)	1.8 (0.9 to 3.5)	0.5 (0.1 to 1.4)	0.4 (0 to 2.7)
*Clostridium difficile*		0.9 (0.3 to 2)	0.2 (0 to 1.3)	0.2 (0 to 1)	0.8 (0.1 to 3.4)

LOS, length of stay; MAU, medical assessment unit.

Although nurses perceived increased falls as a ‘single-room issue’ because of diminished visibility, the temporary nature of the increase in Tunbridge Wells Hospital and lack of a similar pattern in the new-build/mixed accommodation control site suggest that the observed adverse outcomes are not directly associated with single rooms. One explanation might be the disruption from the move to a new environment and the need to adjust work patterns, before patterns of falls settled down again. Our staff interview data suggest some of the necessary adaptations that were made in the 100% single rooms (eg, use of technology, non-slip socks and improved assessment to prevent falls). To minimise falls, the rooms had non-slip flooring and *in-board* bathrooms with bathroom doors on the bed head wall with hand rails (to prevent falls risk by patients walking across an open floor to get to the bathroom). However, as outlined above, the *in-board* bathrooms meant that staff sight lines were limited to one patient.

### Impact on costs

An all-single-room hospital requires more floor space for wards, but space requirements for other areas are the same. Interviews with architects and construction experts suggested that the cost of building an all-single-hospital in the UK is probably no more than 5% higher than a 50% single-room hospital. There was no evidence of difference in maintenance costs per square metre, nor of an increase in the cost of preparing and serving meals related to single rooms. We modelled the cleaning costs for a 500-bed hospital with a 100% and 50% single-room design (table 6). Total annual costs for cleaning ward areas were 53% higher in the all-single-room design and added approximately £1050 per bed per year for cleaning. For a Trust with approximately 500 beds, this cost would be approximately 0.14% of annual budget. We analysed the impact of a number of other costs, such as catering, falls, length of stay and infections.^37^ However, it was impossible to attribute any observed differences to a ‘single-room effect’, and these are not therefore included here.

Comparing nurse staffing data in the 19 months before and after the move up to March 2013, there was an overall increase in whole time equivalent (WTE) for nurses and also a change in the skill mix of staff (a slight increase in proportion of RNs). Overall, under the planned investment at Tunbridge Wells Hospital, there was a 0.9% decrease in beds (from 791 to 784 beds), a 3% increase in WTE staff (from 1793 to 1847 WTE) and a 2.7% increase in total staffing costs (from £60 878 000 to £62 548 000). Attributing how much of the planned investment was the result of a ‘single-room effect’ is, however, not possible. In some areas, including maternity, the changes were part of an overall review of nursing levels and the general restructuring of patient pathways. While there may have been some impact on operational costs arising from increased numbers of staff, this is partially due to the change in number of beds, the cost of staff time and change in the skill mix of staff. There is an opportunity cost associated with time spent walking; this time is not available for other activities such as spending time with patients or administrative activities.

**Table 6:** Impact on costs

Cost item	Single-bedroom hospital
Space requirement	More space required for wards but not for other areas
Building costs per bed	5% higher in 100% single-bedroom hospital than in a 50% single-room hospital
Cleaning costs per bed	53% higher in single bedrooms (extra £1050 per bed per year)
Number of beds	Decreased from 791 to 784 (−0.9%)
Nursing staff WTE	Increased from 1793 to 1847 WTE (3%)
Nursing staff costs	Increased from £60.88 million to 62.55 million (2.7%)

WTE, whole time equivalent.

## Discussion

A disadvantage of single rooms for approximately a third of patients was a sense of isolation and loneliness confirming a recent survey in Scotland.^38^ Patients missed opportunities for interaction with other patients, confirming previous findings.^7^ ^9^ ^23^ Patients and staff noted the potential for loneliness, boredom, loss of shared experience and absence of distraction and social interaction offered by multi-bedded wards. Dayrooms were rarely used, and patients were only orientated to their rooms. However, on balance, most patients in our study preferred single rooms. Comfort, control, privacy and en-suite bathrooms were important aspects that influenced this preference.

Nurses needed to adapt their working practices significantly and felt ill prepared for working on single-room wards, resulting in trial and error of new approaches to care. Staff walking distances increased, and a number of aspects of care, including providing adequate surveillance, were challenging. Staff preference remained for a mix of single rooms and bays, and our findings suggest that a move to all single rooms may have significant implications for the nature of teamwork in the longer term, confirming evidence that suggests 100% single-room facility design made team communication and patient monitoring difficult, and that it limited social interaction among staff.^39^

Our findings suggest single rooms have implications for teamwork and informal learning (ie, observing how colleagues handle situations/role modelling), something not previously identified in the literature. Future research is needed to explore different room designs and ward layouts and consider the long-term impact of single-room working on staff, including the nature of teamwork and informal learning.

Staff interviews highlighted concern about loss of panoptic surveillance of patients, which was seen as a major disadvantage of the new wards, and linked to the perception that falls had increased because of single rooms. While our results suggested that any increases in falls could be explained by changes in case mix, previous evidence suggests single rooms could mean less surveillance by staff and increased rates of slips, trips and falls^40^ ^41^ and falls that are unnoticed by staff or other patients.^8^ ^9^ ^21^ Restricted line of sight into the room and the linear ward layout may have increased walking by nursing staff and reduced efficiency, which confirms other research.^42^ With a different design (eg, observation points and large glazed windows and doors), such adverse effects on staff-to-patient observation might be mitigated.^9^ Design features of wards with single rooms and technology need to be maximised to ensure that privacy and comfort for patients does not compromise staff's ability to monitor them.

The temporary nature of falls increases, and lack of a similar pattern in the control wards, which also experienced an increase in single rooms, suggests that it is not an inevitable result of single rooms. This conclusion must also be interpreted in the light of an overall increase in falls at the hospital level associated with changing patient-level risk factors (an increase of comorbidities associated with service reconfigurations). While we did see a sustained increase in falls in the older people's ward, a strong correlation between changes in the fall rate and in patient-level risk factors associated with service reconfigurations makes it difficult to conclude that single rooms are the cause. However, the possibility that some groups, such as older people, are adversely affected cannot be discounted.

The discrepancies between patient and staff views in terms of preference for single rooms are notable; on balance, two-thirds of patients we interviewed preferred single rooms while only 18% of staff indicated a preference for 100% single rooms. Most staff said they would prefer a mix of single rooms and multi-bedded accommodation, to allow them more panoptic surveillance of very sick and frail elderly patients in particular. A mix of accommodation that allows flexibility or accommodation that can be changed to adapt to patient needs, with sliding glass walls for example, may be a solution. Key for patients was access to an en-suite bathroom, which for many tipped the balance in favour of single rooms, despite some patients experiencing loneliness and isolation. If future innovation in single-room design can enhance visibility for staff, while maintaining access to en-suite bathrooms and facilitate more communal spaces for patients to interact, then staff and patient preferences may become more closely aligned.

### Strengths and limitations

Studying one specific hospital in depth through four case study wards provides rich in-depth data generating important learning for future new hospital builds. Sampling four different wards provides a range of ages; conditions, hospital length of stay and a range of contexts in which to understand staff work experiences and patient experiences of care. However, we were not able to interview patients who were very sick or who had dementia or delirium and we did not interview carers/relatives.

The 152-item survey was designed specifically for this study. Apart from the adapted Teamwork and Safety Climate Survey and some staff well-being questions from the NHS staff survey, the survey was, however, not standardised. Items were generated by reviewing the literature and a pool of potential items were reviewed and revised by a group of health services researchers and pilot tested with a group of 20 nurses. Our controlled before-and-after design provides weak causal inference, but our cautious approach—use of SPC charts within units followed by close scrutiny of other sources of data, including case mix, to assess whether cause was plausible and likely—means that we are unlikely to make incorrect attributions. This is in stark contrast to most previous studies where simple before-and-after comparisons have been used. We used aggregated routine data without individual risk adjustment for patient safety because of the available data (monthly reports of rates), and so a regression-based analysis with individual risk adjustment was not possible. While such routine data are problematic because of coding and reporting bias, our approach to analysis, a time-series-based approach at unit level, using SPC charts, was most appropriate for the data we were able to collect^43^ and protected against many potential biases.

We could not make a causal link between single-bed rooms and changes in the rate of falls, so the cost of falls was not included in the economic analysis, although we recognise that these costs are potentially high. As noted above, differences in healthcare resourcing, cultural and workforce expectations and organisational systems mean it should not be assumed that our findings are necessarily generalisable to other countries.

## Conclusion

This study is one of very few to have examined in depth the experiences of patients and staff in single-room hospital accommodation and provides valuable evidence to guide policy, planning and hospital design. Based on our data, there appear to be no strong economic or safety reasons for choosing 100% single rooms in the UK. Our findings suggest it would be beneficial for managers planning a similar move to encourage staff to prepare and rehearse for working in single rooms, to plan for a possible increase in nurse staffing, to review and reinforce infection control and falls policies for single-room working and to actively manage and monitor changes in work practices and inpatient experiences before and after any changes. Nurses in our study preferred a mix of single rooms and shared patient accommodation. The majority of patients in our study preferred single rooms, but consideration needs to be given to the minority of patients who experienced isolation in single rooms as well as those very sick patients and frail older people, including those with dementia and delirium whom we did not interview. The possibility that some groups, such as frail older people, are adversely affected cannot be discounted. It is therefore important to give careful consideration to the likely patient population when thinking about the mix of accommodation types in other new hospital builds.

## Supplementary Material

Web supplement
