# Supplementary material for: One size fits all? Mixed methods evaluation of the impact of 100% single-room accommodation on staff and patient experience, safety and costs
Source: BMJ Qual Saf. 2015 Sep 25;25(4):241–56. doi: 10.1136/bmjqs-2015-004265 (PMC4819646; doi:10.1136/bmjqs-2015-004265)
Supplement: Web supplement [file bmjqs-2015-004265-s1.pdf]

SUPPLEMENTARY APPENDIX

Organisational context key stakeholder interviews

Table 7: Number of key stakeholder interviews, sampling and topics

Phase	Senior trust staff	New hospital development team	PFI consortium	Senior Medical and AHP staff	Ward managers/ administrator	Sampling	Topics
Pre move (n=20)	8	9	3			Purposive, snowballing	Factors influencing decision to build<br>Management of change process<br>Perceived advantages and disadvantages
Post move (n=21 + 5 in focus group)	8			8 + 5 in mini focus group	5	Purposive, snowballing	Implementation<br>Early challenges<br>Experience across the trust

Details of case study methods and samples

1. Observation of practice

Table 8: Numbers of staff observed and hours of observation by ward and staff type pre and post move

**Staff group**	Phase	Hours observation (no. staff shown in brackets)				**Total**
		Acute assessment	*Medical (older people)*	*Surgical*	*Maternity*	
Nurses/midwives	Pre	23 (3)	13.5 (2)	19.25 (3)	14.75 (3)	**70.5 (11)**
Nurses/midwives	Post	27.4 (5)	23.2 (4)	23.2 (4)	22 (4)	**95.8 (17)**
Healthcare assistants	Pre	6 (1)	15.25 (2)	12.5 (2)	14.5 (3)	**48.25 (8)**
Healthcare assistants	Post	5.6 (1)	8.5 (2)	10.5 (2)	11.2 (2)	**35.81 (7)**
**Total**		**62 (10)**	**60.45 (10)**	**65.45 (11)**	**62.45 (12)**	**250.36 (43)**

Box 1. Task activity categories

1. Direct care
2. Documentation
3. Escort/transfer patient
4. Indirect care
5. Medication tasks
6. Personal/social
7. Professional communication
8. Ward related

2. Staff travel distances

Table 9: Pedometer data collection by staff type, ward and pre and post move

**Staff group**	Phase	Hours pedometer data (no. staff shown in brackets)[1]				**Total**
		Acute assessment	*Medical (older people)*	*Surgical*	*Maternity*	
Nurses/midwives	Pre	111 (11)	54 (5)	74 (8)	40 (5)	**279 (29)**
	Post	58 (9)	27 (7)	59 (8)	33 (6)	**177 (30)**
Healthcare assistants	Pre	30 (1)	118 (11)	49 (6)	39 (6)	**236 (24)**
	Post	24 (5)	62 (12)	54 (7)	11 (2)	**151 (26)**
Total	Pre	141 (12)	171 (16)	123 (14)	79 (11)	**514 (53)**
	Post	81 (14)	89 (19)	113 (15)	46 (8)	**328 (56)**
**Grand total**	**All**	**222 (26)**	**260 (35)**	**236 (29)**	**124 (19)**	**842 (109)**

3. Staff surveys

Table 10: Staff pre and post move survey components

**Scale**	**Format**	**Number of items**
Adapted Teamwork and Safety Climate Survey (Hutchinson et al, 2006)	Likert scales	24
Perceptions of **current** ward layout, environment, facility and information communications technology	5 point Likert scales	35
Improvements needed to **current** environment	Open ended	2
**Perceptions** of single rooms vs multi-bed accommodation – in relation to facilities and for various staff and patient activities[1]	5 point Likert scales	23
Most looked forward to/most liked and least looked forward to/least liked	Open ended	2

features of 100% single rooms[2]<br><br>Preference for ward layout[1]	 <br><br>Categorical	 <br><br>1
Most recent shift activities and satisfaction with care	5 point Likert scale<br>Categorical	18<br>9
Job satisfaction	5 point Likert scale	1
Well being and stress	Categorical<br>Likert scale	5<br>3
Demographic	Categorical	10

[1]**Note:** Questions about perceptions of single vs multi bedded wards were asked pre and post move

[2] **Note:** Pre move questions were about most and least looked forward to features of single rooms. Post move questions were about the most and least liked features

Table 11: Survey response rate before and after the move

Phase	Total population sampled	Response rate
Pre move	176	31%
Post move	204	27%

Table 12: Number of survey respondents by ward

**Phase**	**Ward**				**Total**
	**Maternity**	**Surgery**	**Older people care**	**Acute Assessment**	
Pre Move	17	15	11	12	55
Post Move	26	14	11	4	55
Total	43	29	22	16	110

Table 13: Number of survey respondents by staff type

Phase	**Healthcare assistants**	**Registered nurses/midwives**	**Total**
Pre Move	16	39	55
Post Move	16	39	55
Total	32	78	110

4. Staff interviews

Box 2. Interview topics

Staff experience – working differently / new ways of working

Ward layout including layout of single rooms and en suites

Staff communication and teamwork

Perceptions of patient experience

Table 14: Numbers of staff interviewed by staff group and pre and post move

**Staff group**	Phase	Staff interviews (photo elicitation in brackets)				**Total**
		Acute assessment	*Medical (older people)*	*Surgical*	*Maternity*	
Nurses/midwives	Pre	5 (3)	2 (2)	4 (2)	5 (3)	**16 (10)**
	Post	5 (3)	3 (1)	6 (3)	5 (2)	**19 (9)**
Healthcare assistants	Pre	1 (0)	4 (1)	2 (1)	1 (0)	**8 (2)**
	Post	1 (0)	2 (1)	1 (1)	1 (1)	**5 (3)**
Total	Pre	6 (3)	6 (3)	6 (3)	6 (3)	**24 (12)**
	Post	6 (3)	5 (2)	7 (4)	6 (3)	**24 (12)**
**Grand total**	**All**	**12 (6)**	**11 (5)**	**13 (7)**	**12 (6)**	**48 (24)**

5. Patient interviews

Box 3. Patient interview topics

Recent experience as a patient

Experience of being admitted

Feeling comfortable

Feeling safe

Interaction with staff

Interaction with visitors

Suggestions for improvements

Table 15: Numbers of patients interviewed pre move

Key characteristics		Case study ward				Total
		Acute assessment	Medical (older people)	Surgical	Maternity	
Length of stay (range)		24-48 hrs	2-6 weeks	24 hrs -14 days	24 hrs 5 days	-
Mean age		57 years (range 42-73)	87 years (range 83-94)	58 years (range 19-77)	Not collected	
Parity[1]	Primiparous	-	-	-	5	
	Multiparous				5	
Female		3	2	6	10	21
Male		2	2	7	-	11
TOTAL (interviews per ward)		5	4	13	10	32

Table 16: Numbers of patients interviewed post move

Key demographics		Ward				**Total**
		Medical Assessment Unit (MAU)	*Older people*	*Emergency Surgery*	*Postnatal*	
Length of stay (range)		24 hrs – 7 days	6 days – 5 weeks	24 hrs – 9 days	48 hrs – 7 days	**-**
Mean age		62 years (range 44-74)	82 years (range 70-95)	66 years (range 45-84)	35 years (range 26-49)	
Parity[1]	Primiparous	NA	NA	NA	5	
	Multiparous				3	
Female		4	4	3	8	**19**
Male		4	4	5	NA	**13**
**TOTAL (interviews per ward)**		**8**[2]	**8**	**8**	**8**	**32**

Quasi experimental study of safety outcomes

Table 17: Characteristics of study wards in intervention **and control sites**

	Acute Assessment			Older People		
	Tunbridge Wells	Mixed Accommodation	Steady state	Tunbridge Wells	Mixed Accommodation	Steady state
n	17457	4948	24747	1600	1779	1580
Age	57.5	52.8	64.8	84.4	80.5	82.9
LOS	1.3	1.1	1.5	27.4	12.9	13.8
Charlson	8.2%	4.3%	9.5%	11.8%	4.9%	16.5%

	Surgical		
	Tunbridge Wells	Mixed Accommodation	Steady state
n	4938	1821	8388
Age	60.7	55.0	50.2
LOS	7.2	8.1	1.9
Charlson	10.6%	9.4%	4.1%

Table 18: Data sources and definitions for outcomes, matching and risk stratification and staffing variables

Variable	Source	Definition
		Outcome
Falls	Incident Reports	(Falls per month per ward/bed days)*1000
Pressure Ulcer	Incident Reports	(Pressure ulcers per month per ward/bed days)*1000
Medication error	Incident Reports	(Medication errors per month per ward/bed days)*1000
MRSA	Infection Control	(MRSA cases per month per ward/bed days)*1000
Clostridium difficile	Infection Control	(C. diff. cases per month per ward/bed days)*1000
		Matching/Risk stratification
Age	Administrative	Mean
Length of stay	Administrative	Mean
Primary ICD-10	Administrative	10 most frequent 4-digit ICD-10 codes
HRG	Administrative	5 most frequent HRG sub groups

Charlson Index	Administrative	Percentage of diagnoses included in the Charlson index
Bed days	Administrative	Sum of length of stay per month per ward
		Staffing
WTE All nursing staff	Finance	Total nursing hrs. per patient day (TNHPPD)
WTE Registered Nurses	Finance	Total registered nursing hrs. per patient day (RNHPPD)
WTE Temporary Staff	Finance	Temporary nursing hrs. per patient day (TempHPPD)

Economic analysis

Table 19: Data sources for economic analysis

Data	Source
Construction and operating/maintenance costs	Literature<br>outline business case for new hospital<br>Expert views gathered during interviews<br>healthcare premises cost guides<br>Estate Return Information Collection data over a sample of hospitals<br>Net Present Value of construction costs and lifecycle operating costs discounted 3.5% over the first 30 years and a 3% for the next 30 years to 60 years.
Cleaning costs	Administrative data – three different designs – 100% single rooms, 50% single rooms, 100% multi bedded room – assuming equal numbers of patients
Cost of nurse staffing	Actual monthly WTE for each case study ward<br>Royal College of Nursing data on pay rates to calculate average of all pay scales in each band<br>Data on unit cost of health and social care
Extra nursing time	Time spent in direct contact with patients (observational data)<br>The proportion of contact time was applied to the total nurse hours per patient days (NHPPD) to provide an estimate of the patients contact time per patient day

Table 20: Experts interviewed

Construction management	2
Architectural design in healthcare	4
Strategic estate advisor	1

Hospital facilities management	3
Nursing management	1
Hospital finance management	1
